# Potential role of TGFΒ and autophagy in early crebellum development

**DOI:** 10.1016/j.bbrep.2022.101358

**Published:** 2022-10-03

**Authors:** Azadeh Dalvand, Simone C. da Silva Rosa, Saeid Ghavami, Hassan Marzban

**Affiliations:** Department of Human Anatomy and Cell Science, Max Rady College of Medicine, Rady Faculty of Health Science, University of Manitoba, Winnipeg, MB, Canada

**Keywords:** Cerebellum early development, Autophagy, Autophagy flux, Transforming growth factor-beta

## Abstract

During development, the interconnected generation of various neural cell types within the cerebellar primordium is essential. Over embryonic (E) days E9−E13, Purkinje cells (PCs), and cerebellar nuclei (CN) neurons are among the created primordial neurons. The molecular and cellular mechanisms fundamental for the early cerebellar neurogenesis, migration/differentiation, and connectivity are not clear yet. Autophagy has a vital role in controlling cellular phenotypes, such as epithelial-to-mesenchymal transition (EMT) and endothelial to mesenchymal transition (EndMT). Transforming growth factor-beta 1 (TGF-β1) is the main player in pre-and postnatal development and controlling cellular morphological type via various mechanisms, such as autophagy. Thus, we hypothesized that TGF-β1 may regulate early cerebellar development by modifying the levels of cell adhesion molecules (CAMs) and consequently autophagy pathway in the mouse cerebellar primordium. We demonstrated the stimulation of the canonical TGF-β1 signaling pathway at the point that concurs with the generation of the nuclear transitory zone and PC plate in mice. Furthermore, our data show that the stimulated TGF-β1 signaling pathway progressively and chronologically could upregulate the expression of β-catenin (CTNNB1) and N-cadherin (CDH2) with the most expression at E11 and E12, leading to upregulation of chromodomain helicase DNA binding protein 8 (CDH8) and neural cell adhesion molecule 1 (NCAM1) expression, at E12 and E13. Finally, we demonstrated that the stimulated TGF-β signaling pathway may impede the autophagic flux at E11/E12. Nevertheless, basal autophagy flux happens at earlier developmental phases from E9−E10. Our study determined potential role of the TGF-β signaling and its regulatory impacts on autophagic flux during cerebellar development and cadherin expression, which can facilitate the proliferation, migration/differentiation, and placement of PCs and the CN neurons in their designated areas.

## Highlights

• **TGF-β1** regulates early cerebellar development by modifying the **cell adhesion molecules** and **autophagy flux** in the mouse cerebellar primordium.
• TGF-β1 signaling upregulates the expression of β-catenin and N-cadherin at E11 and E12.
• TGF-β signaling pathway **blocks the autophagic flux** at E11 and E12, however, it does not prevent basal autophagy at earlier developmental phases (E9-E10).
• Cadherin expression can facilitate the proliferation, migration/differentiation, and placement of PCs and the CN neurons in their designated areas.

## 1 Introduction

The cerebellum, also called the “little brain,” is situated as a separate subdivision inferior to the occipital lobe and posterior to the brain stem and fourth ventricle [[1], [2], [3], [4]]. The cerebellum, pons, and medulla oblongata, initially are developed from the rhombencephalon during central nervous system (CNS) embryological growth [5,6]. Around embryonic day E7−E8, the cerebellar primordium appears from the rostral lip of the fourth ventricle in the mouse [[7], [8], [9], [10]]. During development, the cerebellar primordium includes two different germinal zones: the ventrally-situated ventricular zone neuroepithelium and dorsally-situated rhombic lip [11,12]. In mice, the main cerebellar neurons (cerebellar nuclei (CN) and Purkinje cells (PCs)) are created initially on embryonic days E9−E13. The origin of all cerebellar GABAergic neurons such as PCs are from the ventricular zone (between E10−E13), expressing the transcription factor PTF1a, whereas all glutamatergic neurons such as projection neurons of the CN derive from the rhombic lip (between E9-E12) that subsequently may express the transcription factors, for instance, MATH1 and LMX1a [9,13]. Premature cerebellar neurons proliferate, migrate and differentiate from their embryonic position to address the essential cellular event for generating Purkinje cell plate (PCP) and nuclear transitory zone (NTZ) in charge of several molecular pathways [14].

Transforming growth factor-beta (Tgf-β) superfamily members has a crucial role in CNS development [15]. Based on the cell type, the developmental phase/time, and tissue location, may create several biological outcomes [16]. Furthermore, the TGF-β proteins prepare postural information for distinct progenitor cells by creating long-range concentration gradients, resulting in cell fate decisions and tissue patterning [17,18]. In addition, they also function in a spatiotemporal mode and play decisive roles in adult tissue homeostasis and scar generation/injury repair [[19], [20], [21]]. Although in recent years, the impacts of TGF-β family proteins in the CNS have been investigated comprehensively, little information is available regarding their expression and outcome on development and pathological cerebellar episodes [15,22]**.**

Epithelial-to-mesenchymal transition (EMT) is a natural phenomenon, which happens during usual and unusual physiological and pathological episodes [[23], [24], [25]]. EMT has crucial roles, spanning from embryonic development and cellular differentiation to tissue repair [[26], [27], [28]]. In the course of EMT, polarized epithelial cells with specific cell-to-cell interplay and connections with the basement membrane lose their epithelial features and acquire mesenchymal characteristics, such as migratory and invasiveness actions [24,26]. Thus, cells with a new mesenchymal morphological type can relocate from their epithelial nest to close or distant positions [29].

The cadherins, an extensive family of Ca^2+^-dependent transmembrane glycoproteins including more than 100 members [30], are extremely crucial for EMT. As the cell adhesion molecules (CAMs), cadherins contribute to several morphogenetic episodes during development, such as cell-cell attachment and cell migration [31]. According to their sequence similarity, classical cadherins have been categorized into types I and II [30,32]. Classical cadherins are single-pass transmembrane proteins with an extracellular domain including five repeat sequences and two cytoplasmic catenin-binding domains that connect cadherin protein to the actin cytoskeleton meshwork and intracellular signaling pathways [30,33]. These elements provide the strong cell-cell adhesion, intervened by the cadherins [32].

TGF-β provokes phenotypic alterations in the cells via reprogramming of cadherin gene expression [24,31]. It has been indicated that TGF-β treatment of cultured epithelial cells results in stimulation of the TGF-β signaling pathway, such as SMAD proteins [25,29,34]. Moreover, the SMAD4 protein has been determined as an intermediary of TGF-β, a strong regulator of EMT; although the mechanisms connecting TGF-β signaling to cadherin expression are relatively unknown [35]. However, the proliferation of the epithelial cells and expression of the epithelial markers are blocked, and mesenchymal marker expression is elevated, resulting in increased cell motility [26,29].

Macroautophagy (hereafter called autophagy) is a closely controlled lysosomal degradation and clearance pathway [[36], [37], [38]]. This conserved intracellular catabolic pathway provides energy during usual and unusual physiological states by sending cytoplasmic constituents to the lysosome and subsequently degrading them [[39], [40], [41], [42]]. Autophagosome generation is a crucial act in autophagy, requiring the expression of the autophagy-related genes (ATG), including ATG12, ATG5, ATG16L1, and LC3β (ATG8) [[43], [44], [45], [46]]. Furthermore, as a cell autodigestive process, autophagy is extremely involved in cerebellar differentiation and development [10,47,48].

In the course of embryogenesis, autophagy help in fast cellular alterations required for development by reacting to extracellular indications [47]. Thus, as an autorenewal mechanism to control or balance stress conditions, such as starvation, basal autophagy continuously circulates the energy internally in the cell environment and the absence of any particular stimulation [49,50]. Moreover, several studies have indicated that TGF-β_1_ stimulates concurrent autophagy through the SMAD signaling pathway and alteration of phenotype in different cell models like HuH7 human hepatocellular carcinoma cells [51,52] and non-small cell lung cancer (NSCLC) cell lines A549 and H1975 [25].

It is not clear how the extremely complicated cerebellar circuit is wired and compiled during development. Using mice cerebellar primordium, we emphasized the molecular mechanisms elemental to the precise and temporal control of the intracellular signaling network generated by the Tgf-β signaling pathway exchange with other signaling pathways, including cadherins-dependent cell-cell adhesion and autophagy at the primordial embryonic days (E9−E13). We hypothesized that Tgf-β_1_ may regulate early cerebellar development by changing the levels of CAMs and autophagy pathways in the mouse cerebellar primordium. Here, we assessed the expression of principal molecular pathways involved in cerebellar primordium development and autophagy pathway. Furthermore, we demonstrated that Tgf-β_1_ potentially alters cadherins and autophagy flux, assisting in early cerebellar domain creation. Thus, our study paves the way for understanding the role of TGF-β1 during early cerebellar development and may address the concerns regarding neural connectivity and the natural selection of post-mitotic cerebellar neurons.

## 2 Materials and methods

### 2.1 Animal maintenance

All animal procedures followed the institutional regulations and the *Guide to the care and use of experimental animals* from the Canadian Council on Animal Care (CCAC), authorized (protocol # AC11527) by the Bannatyne Campus Animal Care Committee (ACC), at the University of Manitoba, whose mandate is to reduce the number of laboratory animals and the associated experienced discomfort used in test procedures. Animals were acquired from the Central Animal Care Services, Faculty of Medicine, University of Manitoba, Winnipeg, MB, Canada.

### 2.2 Sample collection

**Fig. 1:**
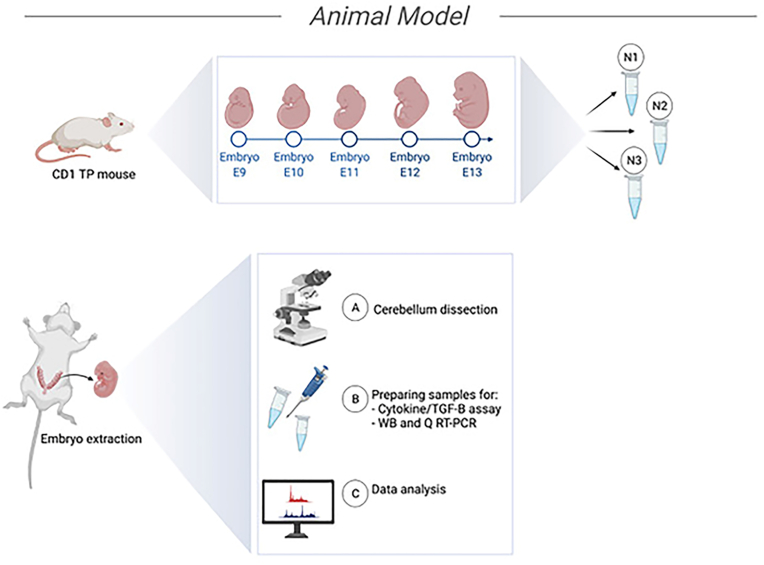
Workflow from embryonic cerebellar primordium sample collection, processing and analysis.

Timed-pregnant CD1 female mice at embryonic days E9 to E13 were deeply anesthetized with 20% isoflurane, and USP (Baxter Co. Mississauga, Ontario, Canada) diluted with propylene glycol (Sigma-Aldrich Canada Co., Ontario, Canada) in a desiccator. Using a set of fine forceps, placed in a Petri dish containing ice-cold 0.1 M phosphate buffer saline (pH 7.4), embryos at chosen embryonic days were separated from the uterus of an impregnated mouse. Subsequently, using a SteREO microscope (Discovery.V8, Carl Zeiss) and tweezers with tapered tips with high precision points (Excelta™ 5SA), ectoderm and meninge were carefully detached. Finally, the cerebellar primordium was removed from the encircling tissues. The harvested tissue pieces were immersed in RNAlater-ICE Stabilization Solution (Ambion, Cat# AM7030) and lysis buffer consisting of NP-40 lysis buffer (150 mM sodium chloride, 1.0% NP-40, 50 mM Tris PH 8.0), protease inhibitor cocktail (Life Science, Cat# M250) and phosphatase inhibitor (Sigma Aldrich, Cat# P5726) to protect cellular RNA and proteins, respectively. Collected samples were kept at −80 °C before processing. Each replicate of samples was aggregated with a minimum of 100 CD1 embryos (Fig. 1).

### 2.3 Multiplex cytokine assay

The collected embryonic cerebellar tissues, immersed in approximately 2 × tissue volume of lysis buffer, were homogenized by sonication and centrifuged at 12,000×*g* for 10 min at 4 °C. Afterward, the supernatant was collected in a new tube. Protein concentrations were examined using a commercial BSA kit (Bio-Rad, Cat# 5000121). Samples were diluted in standard PBS (pH 7.4), as the dilution buffer and stored at −80 °C, following the manufacturer's instruction (Eve Technologies, Calgary, Canada). A 150 μl aliquot of each age was tested with Multiplex LASER bead Discovery assays for three different isoforms of Tgf-β 1, 2, and 3. Each assay was done in duplicate in three independent biological replicates. Active TGF-β1, TGF-β2, and TGF-β3 levels were examined, and the final concentration of 1 μg/μL was chosen as the optimum dilution for all the samples (Eve Technologies, Calgary, AB, Canada). All the assays were repeated in three different replicates for each embryonic day. Data were analyzed using one-way ANOVA. P < 0.05 was deemed statistically significant.

### 2.4 Immunoblotting

The protein analysis of the cerebellar homogenates derived from CD1 mouse embryos at E9 to E13 was done following a conventional protocol [53,54]. Isolated cerebellar tissues submerged in lysis buffer (approximately 2 × of the volume of the tissue) were homogenized by sonication and centrifuged at 12,000×*g* for 10 min at 4 °C. The protein concentration of the supernatant was measured by a BSA kit (Bio RAD laboratories, USA; Cat# 500–0114). Then, samples were prepared for loading with a final concentration of 1 μg/μL, by adding the loading buffer (Tris-Hcl 60 mM, glycerol 25%, SDS 2%, mercaptoethanol (ME) 14.4 Mm, bromophenol blue 0.1%, and H_2_O).

Samples were heated for 10 min at 90 °C before loading on the gel. After cooling down, equal amounts of protein (20 μg) were separated by polyacrylamide gels, using 4% stacking gel and 10–15% resolving gels. Ten microliters of Precision Plus Protein™, all blue standards, were used as protein ladder (Bio RAD laboratories, USA; Cat# 161–0373) and loaded on the gel parallel to experimental samples. Then, proteins were transferred to PVDF membrane (Millipore, Mississauga, ON, USA) in transfer buffer (500 nM glycine, 50 mM Tris-HCl, and 20% methanol) at room temperature (RT) for 2 h at a voltage of 100 V. After blocking the membranes with 5% skim milk (non-fat dried milk in 0.2% TBST (1 × TBS + 0.2% Tween-20) at RT for 1 h, membranes were probed overnight with appropriate primary antibodies (Table 1) in 1% skim milk in 0.2% TBST at 4 °C with gentle agitation. Membranes were then washed three times for 10 min in 0.2% TBST at RT, then kept for 2 h at RT with horseradish peroxidase (HRP)-conjugated secondary antibodies (Table 2) in 1% skim milk in 0.2% TBST. Afterward, blots were rinsed three times for 10 min in 0.2% TBST. Subsequently, the membranes were soaked in the enhanced chemiluminescence (ECL) reagents (1 ml Peroxide Reagent and 1 ml Luminol/Enhancer) (Cat# 170506; Clarity Western ECL Substrate) for 60 s. At last, binding was detected by the BIORAD ChemiDoc Imager and quantified using the dosimetry software Alpha Ease FC.

**Table 1:** Primary antibodies used for Immunoblotting.

Name of Antibody	Dilution	Source
Anti- N-Cadherin	1:500	Developmental Studies Hybridoma Bank Catalog# MNCD2
Anti- NCAM	1:500	Developmental Studies Hybridoma Bank Catalog# 5B8
Anti- CDH8	1:1000	Developmental Studies Hybridoma Bank Catalog# CAD8-1
Anti- βcatenin	1:2000	BD Bioscience Catalog# 610153
Anti- Smad 2/3	1:1000	Cell Signaling Technology Catalog# 5678S
Anti- Psmad 2	1:1000	Cell Signaling Technology Catalog# 18338S
Anti- P62	1:3000	Cell Signaling Technology Catalog# 3912S
Anti- LC3β I/II	1:3000	Sigma-Aldrich Catalog# L8918

**Table 2:** Secondary antibodies used for Immunoblotting.

Name of Antibody	Dilution	Source
HRP conjugated to goat anti-rat IgG	1:5000	Milipore, AP136P
HRP conjugated to goat anti-mouse IgG	1:5000	Milipore, AP308P
HRP conjugated to goat anti-rabbit IgG	1:5000	Milipore, AP307P

### 2.5 RNA extraction & cDNA synthesis

The harvested cerebellar tissue pieces from mouse embryos at E9 to E13 were immediately immersed in RNAlater-ICE Stabilization Solution overnight at 4 °C, then removed from the reagent and kept at −80 °C. As per the manufacturer's instruction, total RNA was extracted and purified using RNeasy Plus Mini Kit (QIAGEN, Cat# 74134 & 74136). Optimal RNA yield and purity were determined by nanodrop ND-1000 UV–Vis Spectrophotometer (Thermo Fisher Scientific, Waltham, MA, USA). The cDNA was synthesized by a qScript cDNA Synthesis kit (Quantabio, Cat# 95048–100), using 1000 pg of RNA.

### 2.6 Real-time PCR

Quantitative reverse transcriptase-PCR was carried out in a final volume of 20 μL, containing 2 μL of cDNA template, 1.2 μL of exon-specific primer pairs (forward & reverse), and 10 μL SYBR Green Master Mix (Applied Biosystems™, Catalog No. A25742) in a 96-well plate [6,55]. The primers are listed in Table 3. All reactions were carried out in duplicate, and β–actin was chosen as a housekeeping gene to normalize gene expression values. Thermal cycling and quantification were done by the real-time PCR (Quantastudio3, Applied Biosystems), using the following cycling conditions: initial denaturation at 50 °C for 2 min, and then 95 °C for 10 min, followed by 40 cycles of 95 °C for 15 s and 60 °C for 1 min. The melt curve analysis was used to determine the specificity of the amplified product, immediately following the completion of the final amplification cycle. Relative gene expression was normalized to the amount of β-actin mRNA in the same cDNA by the comparative Ct (2^–ΔΔCt^) method. All reactions were performed in three independent replicates, and data are presented as mean ± SEM.

**Table 3:** Primer Sequences used for mRNA quantification.

Gene	Forward sequence 5′ to 3′	Reverse sequence 5′ to 3′
*N-Cadherin (cdh2)*	CGG TTT CAC TTG AGA GCA CA	CAT ACG TCC CAG GCT TTG AT
*NCAM*	TGG TTC CGA GAT GGT CAG TT	GGA TGG AGA AGA CGG TGT TG
*cdh8*	TGC TGA CGA CCC AGT TTA AT	GGT TCC AGA CAG ACC ACC AG
*βcatenin*	CAG ATC CCA TCC ACG CAG TT	ATT GCA CGT GTG GCA AGT TC
*TβRI*	ATT GCT GGT CCA GTC TGC TT	CCT GAT CCA GAC CCT GAT GT
*TβRII*	ACT GTC CAC TTG CGA CAA CCA GAA	AGA AGC GGC ATC TTC CAG AGT GAA
*βactin*	CTG TCC CTG TAT GCC TCT G	ATG TCA CGC ACG ATT TCC

### 2.7 Statistical analysis

All tests were repeated in triplicate per embryonic day. The statistical analysis and figure preparations were done by one-way and two-way ANOVA, using Graphpad Prism 8.0.2 statistical software, and *P*-values < 0.05 were considered significant. Results are shown as mean ± SEM.

## 3 Results

### 3.1 Active TGF-βs are present in cerebellar primordium during early developmental stages at E9−E13

**Fig. 2:**
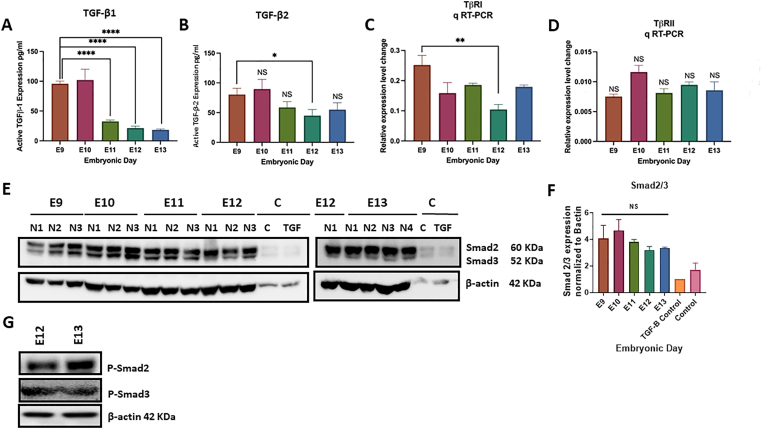

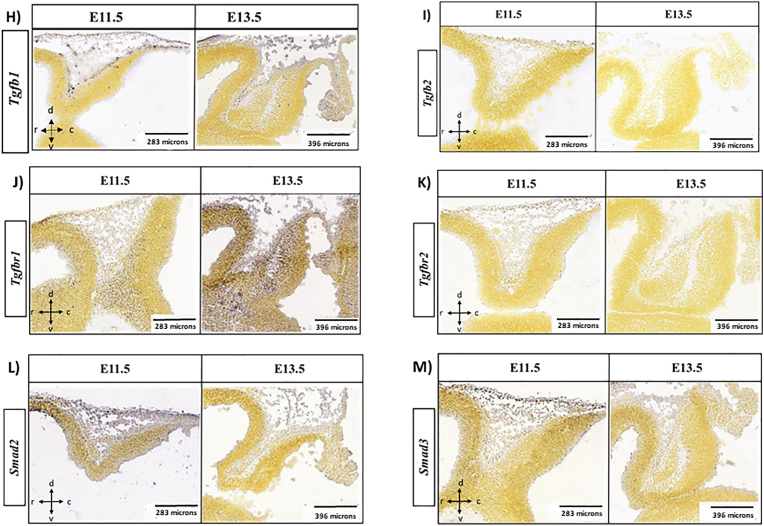
Active Tgfbs and Smad signaling pathway elements changes in cerebellar primordium during early developmental stages at E9-E13 (n = 3). **A, B)** Tgfb1 highly expressed during earliest embryonic days at E9 and E10. Its expression levels decreased significantly with considerable differences from E11 to E13 (**A**). Tgfb2 values experienced almost the same expression pattern as Tgfb1, with higher and lower amounts at E9-E10 and E11-E13, respectively. However, the changing expression pattern of Tgfb2 is not statistically significant (**B**). **C, D**) mRNA expression of Tgfbr1 and Tgfbr2 were measured by RT-qPCR. Tgfbr1 transcription at E9 is higher with a significant reduction at E12 **(C**). mRNA values of Tgfbr2 maintained at the same level with no significant changes from E9- E13 **(D**) (The significance of the receptor expression was compared to E9 and the relative expression was compared to β-actin (universal gene control). **E-G**) Downregulation of total Smad2 & 3 and confirmation of Smad2 and Smad3 protein phosphorylation in E12 and E13. Anti-total Smad2 & 3 reactive bands in Western blot analyses (**E**) and quantification of total Smad2 &3; Total Smad2 and 3 values upregulated at E9 and E10, compared to their correspondent controls, with a non-significant reduction from E11 to E13 **(F**). Anti-phosphorylated Smad2 and Smad 3 showed its phosphorylation in E12 and E13 **(G**). The blots were quantified compared to the TGF-β1 treated cells using densitometry software Alpha Ease FC. The protein loading was confirmed using B-actin. The data in the bar graphs are presented as the mean ± SEM, and statistical analysis was performed using one-way ANOVA (P-Value > 0.05, considered as non significant, P-value ≤ 0.05 and lower were considered statistically significant. ** shows P < 0.01, **** shows P < 0.0001). **H-M**) Distribution of the *Tgfb1 & 2, Tgfbr1 & 2, and Smad2 & 3* in the cerebellar primordium at E11.5, and E13.5. All images show cerebellar primordia and image credit: Allen Institute. © 2008 Allen Institute for Brain Science, Allen Developing Mouse Brain Atlas. Available online at: https://developingmouse.brain-map.org/. **H**) *Tgfb1* was only detected at E11.5 in pia mater of the cerebellar primordium and very low level in the neuroepithelium and tela choroidea. **I**) ISH patterns of *Tgfb2* on sections at E11.5, and E13.5 were not detectable. **J**) Sagittal sections show dynamic expression of *Tgfbr1* express at both E11.5 and E13.5 which is detected in nuclear transitory zone (NTZ) at E11.5 with higher levels of expression in the NTZ and Purkinje cell palate at E13.5. **K**) *Tgfbr2* expression is not detected at any given time points. **L**) *Smad2* expression is strongly high throughout the whole cerebellar primordium at E11.5. No expression is detected at E13.5. **M**) *Smad3* has moderate expression in the cerebellar primordium mainly in the Purkinje cell plate and rhombic lip of the cerebellar primordium at E11.5, but not at E13.5.

Protein profiling of Tgf-β demonstrated that among the three isoforms Tgf-β1, Tgf-β2, and Tgf-β3, the two isoforms of Tgf-β1 and Tgf-β2 are present in the mouse embryonic cerebellar tissue at E9 to E13. The data analysis showed the activated forms of both Tgf-β1 and Tgf-β2, while an absence of evidence was found for Tgf-β3 during early cerebellar development (no Tgf-β3 detected) (Fig. 2A and B). The key finding is that active Tgf-β1 was significantly greater at primordial embryonic days (both E9 and E10), followed by a significant (p ≤ 0.05) decrease from E11 to E13 (Fig. 2A). Active Tgf-β2 revealed the same trend as Tgf-β1, with higher active levels at E9 and E10 and a non-significant reduction in E11 and E13. The Tgf-βr1 (TGF-β receptor 1) and Tgf-βr2 expression were also validated using RT-qPCR at E9−E13 period, in the cerebellar primordium (Fig. 2C and D). Our data showed the expression of the receptrs in E9-E13 (Fig. 2C and D). The significance of the receptors expression were compared to E9 and the relative expression were compared to β-actin (universal gene control). Our data emphasize the potential role of Tgf-β1 during the early developmental phase of the cerebellar primordium, demonstrated by the higher expression of this cytokine at E9 and E10. We also indicated that Smad2/3 and activated Tgf-β1-induced Smad signaling pathway, are present in the cerebellar primordium during early cerebellar development (Fig. 2E–G).

RNA *in situ* hybridization (ISH) data was used to determine the specific localization of the *Tgfb1 and Tgfb2,* RNA nucleic acid sequences, and gene expression profiling in the cerebellar primordium using “Allen Developing Mouse Brain Atlas (DMBA)” (RRID:nif-0000–00509) (http://developingmouse.brain-map.org). The Allen DMBA comprises spatial and temporal gene architecture of the brain development that analyzes gene expression and detection of localization of RNA sequences in the series of sagittal sections through the entire embryo or brain, by RNA probes. ISH data analysis in Allen DMBA are classified into embryonic and postnatal mouse brain developmental time points, including embryonic days E11.5, E13.5, E15.5, E18.5, and postnatal days [56,57]. In our study, considering the given embryonic days (E9−E13), we used ISH data analysis at E11.5 and E13.5 to determine the gene expression following the anatomical zones of the cerebellar primordium.

The spatial-temporal gene expression pattern of *Tgfb1* demonstrates its punctate RNA expression at E11.5 in the pia mater, tela choroidea, and an insignificant amount in the neuroepithelium of the cerebellar primordium (Fig. 2H). Nevertheless, the *Tgfb1* expression is not found by RNA probes at E13.5 in sagittal sections through the cerebellar primordium, only found at a low quantity in the pia mater and choroid plexus of the fourth ventricle (Fig. 2H). The *Tgfb2* RNA expression at both E11.5 and E13.5 time points is indistinct in the cerebellar primordium (Fig. 2I).

Expression of downstream signaling elements of the canonical Tgf-β signaling pathway, including Tgf-βr1 and Tgf-βr2, and Smad2 and 3 transcription factors at E11.5 and E13.5 time points were confirmed by the Allen DMBA. ISH patterns for *Tgfbr1* and *Tgfbr2* in sagittal sections during early cerebellar development revealed the *Tgfbr1* dynamic expression at both E11.5 and E13.5 (Fig. 2J and K). The *Tgfbr1* moderate expression was detected in NTZ at E11.5 (Fig. 2J). The *Tgfbr1* expression altered and highly increased at E13.5, which are identified across the whole cerebellar primordium, primarily placed in the NTZ and PCP (Fig. 2J). Contrarily, no *Tgfbr2* expression was found at any given time point in the sections of the developing mouse cerebellum (Fig. 2K).

Using the Allen DMBA database, we demonstrated that the *Smad2* expression is elevated at E11.5 across the cerebellar primordium, while its expression is almost diminished at E13.5, validating our Western blotting data (Fig. 2L). ISH data showed a moderate *Smad3* expression in the cerebellar primordium, principally in the PCP at E11.5. Like *Smad2*, the *Smad3* expression is reduced at E13.5 (Fig. 2M).

### 3.2 Cadherins’ expression is increased in cerebellar primordium during early developmental stages at E9−E13

#### 3.2.1 Tgf-β signaling pathway

**Fig. 3:**
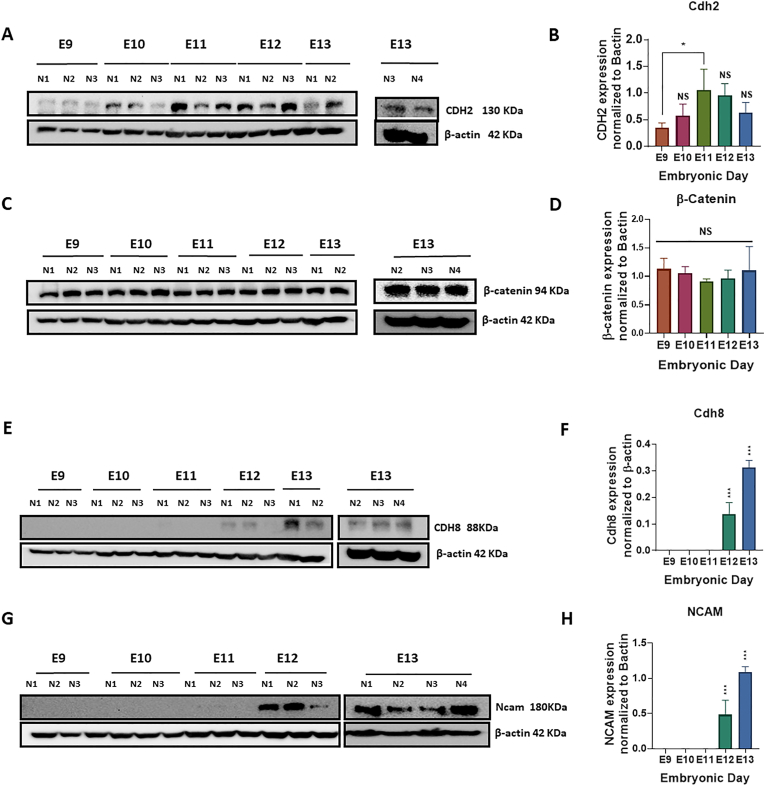

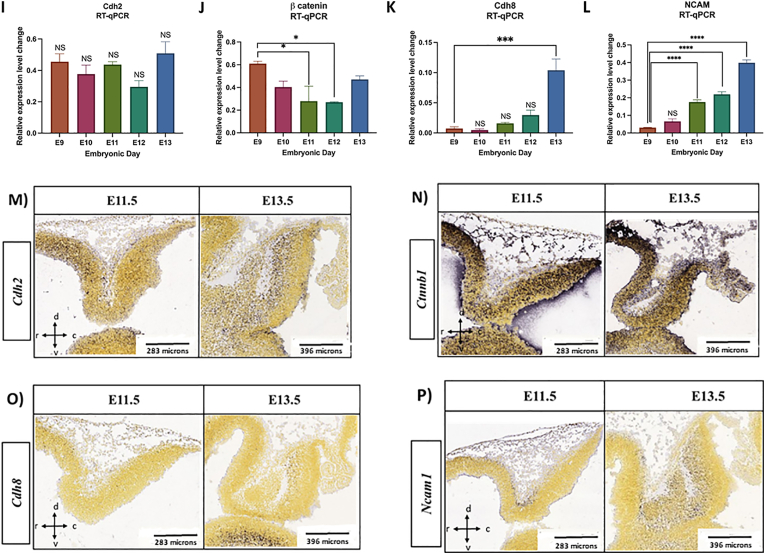
Cadherins expression are upregulated in cerebellar primordium during early developmental stages at E9-E13. **A-H**) Expression levels of Cdh2, Ctnnb1, Cdh8 and Ncam1 proteins measured by Western blotting in mice cerebellar primordium at E9-E13 (n = 3). The protein loading was confirmed using β-actin. **A, B**) Anti**-**Cdh2 reactive bands in Western blot analyses and quantification of Cdh2; Protein expression is upregulated from E9 to E11 and reached its higher amount at E11, which is statistically significant. **C, D)** Western blot analyses and quantification of Ctnnb1; protein expression remained unchanged with no significant differences between embryonic days. **E, F**) Cdh8; protein blots show no expression during the earliest embryonic days at E9-E11. Cdh8 protein expression is upregulated from E12 to E13 and reached its higher amount at E13, which is statistically significant. **G, H**) Data show no Ncam1 expression during the earliest embryonic days at E9-E11. Ncam1 protein expression is upregulated from E12 to E13 and reached its higher amount at E13 which is statistically significant. **I-L**) mRNA expression levels of *Cdh2*, *Ctnnb1*, *Cdh8* and *Ncam1* measured by RT-qPCR in cerebellar primordium during early developmental stages at E9-E13 (n = 3). **I**) *Cdh2* mRNA expression remained unchanged with constant level of expression during the earliest embryonic days at E9-E13. **J**) The trend for *Ctnnb1* mRNA values is negative; transcription at E9 is higher with significant reduction at E11 and E12. **K**) *Cdh8* mRNA expression level experienced an upward trend from E9 to E13, with lower expression levels at E9/E10, which is significantly increased at E13. **L**) *Ncam1* mRNA levels increased from E9 to E13 with lower and higher levels of expression at E9 and E13, respectively. The data in the bar graphs are presented as the mean ± SEM, and statistical analysis was performed using one-way ANOVA (P-value ≤ 0.05 and lower were considered statistically significant. * shows P < 0.05, ** shows P < 0.01, *** shows P < 0.001, **** shows P < 0.0001). **M-P**) Distribution of the *Cdh2*, *Ctnnb1*, *Cdh8* and *Ncam1*in the cerebellar primordium at E11.5, and E13.5. All images show cerebellar primordia and image credit: Allen Institute. © 2008 Allen Institute for Brain Science, Allen Developing Mouse Brain Atlas. Available online at: https://developingmouse.brain-map.org/. **M**) *Cdh2* ISH data shows constant expression of Cdh2 mRNA at E11.5, and E13.5, with condense pattern in the nucmear transitory zone and Purkinje cell plate (mostly in Foxp2^+^ cells) at E13.5. **N**) ISH data shows enrich expression of *Ctnnb1* in the entire cerebellar primordium at E11.5 which is localized in the Purkinje cell plate at E13.5 (less in nuclear transitory zone). **O**) *Cdh8* mRNA is not detected at E11.5 but is detectable in caudal part of the Purkinje cell plate at E13.5. **P**) *Ncam1* ISH data shows strong expression pattern in the nuclear transitory zone of the cerebellar primordium at E11.5, and both nuclear transitory zone and Purkinje cell plate at E13.5.

Further, we attempted to learn about the potential regulatory action of the TGF-β signaling pathway through its interplay with other pathways engaged in extracellular matrix (ECM) protein biosynthesis and development. Thus, we assayed both mRNA and protein expression levels of CAMs, such as *N-Cadherin/Cdh2*, *Cdh8*, *Ncam1*, and β-Catenin (*Ctnnb1*), to demonstrate if there is any correlation between *Tgf-β*_*1*_ and their expression, and possible involvement of *Tgf-β*_*1*_ in their regulation during early cerebellar development. Our data revealed an opposite association between Tgf-β_1_ and the CAM expression in cerebellar primordium within five embryonic days **(**Fig. 2, Fig. 3). We noted a decreased tendency in *Tgf-β*_*1*_ and a growing tendency in the CAMs' expression levels. These data approve the idea that the elevated *Tgf-β*_*1*_ expression during embryonic days E9 and E10 potentially stimulates the canonical *Tgf-β* signaling pathway, transfers the signal to the nucleus via the SMAD family of transcription factors, upregulates the CAMs’ expression, and ultimately controls the neural migration and differentiation processing during cerebellar primordial development.

#### 3.2.2 N-cadherin/Cdh2 signaling pathway

**Fig. 4:**
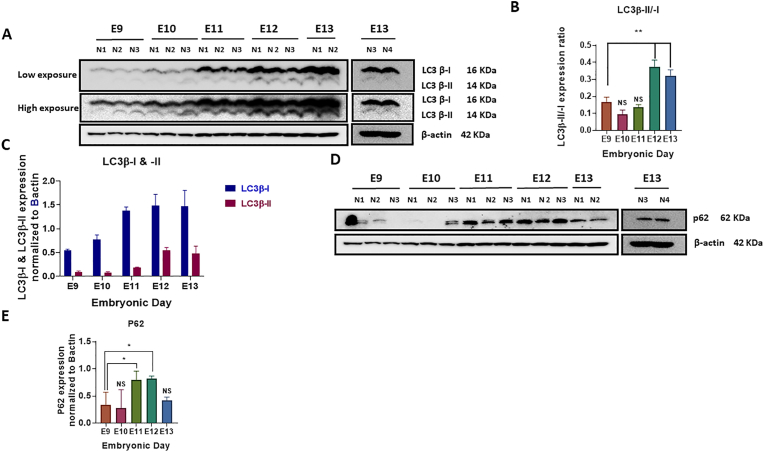
Autophagic-flux is potentially inhibited in cerebellar primordium during early developmental stages at E9-E13. **A-E**) LC3β I & II and p62 protein expression levels measured by Western blotting in cerebellar primordium at E9-E13. **A**) Anti-LC3β-I & II reactive bands in Western blot analyses and **B**) quantification of LC3β-I & II; protein expression levels increase by time; however, LC3β-II value is lower than LC3β-I at each embryonic day. **C**) Quantification of LC3β-II/LC3β-I ratio increase gradually from E9 to E13, and the values reached the highest levels at E12 and E13, which is statistically significant compared to E9. **D**) Anti-P62 reactive bands in Western blot analyses and **E**) quantification of P62; protein expression levels experienced an upward trend from E9 to E12, followed by a rapid reduction at E13. The P62 value is statistically significant at E11 and E12 compared to E9. The blots were quantified using densitometry software Alpha Ease FC. The protein loading was confirmed using β-actin. Data in the bar graphs are presented as the mean ± SEM, and statistical analysis was performed using one-way ANOVA (P-value ≤ 0.05 and lower were considered statistically significant. * shows P < 0.05, ** shows P < 0.01).

For expression profiling of N-cadherin/CDH2*,* the extraction of protein and mRNA from mouse embryonic cerebellar tissues was performed and Western blotting and RT-qPCR were carried out (Fig. 3). An increasing trend was noted in the CDH2 protein expression starting from E9, reaching uppermost expression in E11. As a consequence, the CDH2 expression showed a statistically insignificant declining trend from E12 to E13 (Fig. 3A and B). No statistically significant *Cdh2* mRNA time-dependent expression was found between E9 to E13, and the expression level was constant with minor variation from E9 to E13 (Fig. 3I). These data propose a continuing action of the *Cdh2* gene across five embryonic days from E9 to E13. In comparison with Western blot data, with an increase in total protein expression from E9 to E11, the constant *Cdh2* mRNA expression proposes potential translation enhancers between embryonic days 9–11 in the cerebellar primordium (Fig. 3A, B, I). In addition, it shows that Cdh2 proteirn expression is not only regulated with gene expression but also probably with translation regulation and balance of degradation and biosynthesis (based on the changes on autophagy flux during the early development, Fig. 4) during the cerebellar development. ISH patterns of *N-Cadherin/Cdh2* on sagittal sections throughout the cerebellar development confirm the persistent *Cdh2* expression during the stated time points at E11.5 and E13.5 (Fig. 3M). As illustrated, the *Cdh2* expression changes from diffuse expression across the entire cerebellar section at E11.5 to the compact pattern in the NTZ and PCP (mostly in FOXP2^+^ cells) [58] of the cerebellar primordium at E13.5 (Fig. 3M).

#### 3.2.3 β-Catenin (Ctnnb1) signaling pathway

Further, the evaluation of Ctnnb1 expression level in mouse embryonic cerebellar tissue at E9−E13 revealed that the Ctnnb1 protein expression stayed at the same rate without any significant alteration over embryonic days (Fig. 3C and D). The quantification of the extracted mRNA samples from embryonic cerebellar tissues by RT-qPCR (Fig. 3J) showed a downward trend for the *Ctnnb1* transcription with the highest expression level at E9 and a significant decrease at E11 and E12 (Fig. 3J). In addition, the mRNA level demonstrated a moderate increase at E13, which was not significant (Fig. 3J). The results showed that Ctnnb1 proteirn expression is not only regulated with gene expression but also with translation regulation and balance of degradation and biosynthesis during the cerebellar development. The Allen DMBA results demonstrated a robust *Ctnnb1* expression at both E11.5 and E13.5. The high expression can be demonstrated in the entire sagittal sections through the cerebellar primordium at E11.5 and located in the PCP at E13.5 (less in NTZ) (Fig. 3N). The ISH data regarding *Ctnnb1* validate the Western blot and RT-qPCR results, indicating steady Ctnnb1 protein and mRNA expression levels during the initial embryonic days from E9 to E13 (Fig. 3C–D, J, N).

#### 3.2.4 Cadherin 8 (Cdh8) signaling pathway

To assess the expression pattern of CDH8 in mouse embryonic cerebellar tissues, Western blot analysis was done. No CDH8 expression was found over the development of the cerebellar primordum days from E9−E11 (Fig. 3E and F). The highest expression level was observed at E13 (p < 0.001), starting at E12 (Fig. 3E and F). The RT-qPCR data show, *Cdh8* transcripts on all embryonic days from E9 to E13 (Fig. 3K). Along the lines with the pattern of protein increase, the *Cdh8* mRNA expression increased progressively, from the lowest at E9, to significantly higher at E13 (Fig. 3E–F, K). This positive development demonstrates an enhancement of the *Cdh8* mRNA transcript, accompanied by CDH8 protein expression in cerebellar primordium at E12−E13. No *Cdh8* mRNA was detected based on ISH data at E11.5 but the expression was identified in the caudal part of the PCP at E13.5 (Fig. 3O**)**.

#### 3.2.5 Neural cell adhesion molecule 1 (Ncam1) signaling pathway

NCAM1 as the notable neural CAM is expressed on the surface of early embryonic neurons and glia. NCAM1 plays a key role in facilitating adhesion among neurons and neurite branches. A highly significant Ncam1 protein expression was found (p < 0.05) at E12−E13 (Fig. 3G and H)). Our RT-qPCR data demonstrated a rising trend in *Ncam1* mRNA expression in cerebellar primordium across five embryonic days (Fig. 3G–H, L). A distribution of *Ncam1* was found in the cerebellar primordium at both E11.5 and E13.5, based on ISH data (Fig. 3P). The *Ncam1* expression can be observed in the sagittal cerebellar section in the NTZ at E11.5. Moreover, ISH demonstrated a high *Ncam1* expression in the NTZ and PCP at E13.5 (Fig. 3P). These data validate our Western blot (Fig. 3G and H) and RT-qPCR results, showing an increase in mRNA and protein expression gradually from E9 to E13 (Fig. 3L).

### 3.3 Autophagy flux is potentially inhibited in cerebellar primordium during early developmental stages at E9−E13

Autophagy, as a highly conserved lysosomal degradation and clearance pathway, is required for proper proliferation/migration, differentiation, development, remodeling, and intracellular refreshment [[59], [60], [61]]. An autophagosome marker, namely microtubule-associated protein light chain 3 beta (LC3β) contributes to the generation or degradation of autophagosomes [[62], [63], [64]]. LC3β comprised of LC3β-I and LC3β-II with a molecular weight of 16 kDa and 14 kDa, respectively, are detected in Western blot. Furthermore, LC3β-I is a cytosolic protein, and LC3β-II is positioned on the autophagosome membrane, demonstrating the generation or degradation of autophagosomes [[65], [66], [67]]. The LC3β-II:LC3β-I ratio was applied to determine the LC3 conversion as an autophagosome marker, used to monitor autophagy. In agreement with our data, the LC3β-II:LC3β-I ratio showed a significant rising trend from E9 to E13 (Fig. 4A and B). This proposes an increase in autophagosome creation or a decrease in autophagosome degradation [67,68].

Further, we examined the abundance levels of LC3β-I and –II distinctly. Whereas an increasing trend was found for both LC3β-I and –II expression levels, LC3β-II is lower than LC3β-I at each embryonic day (Fig. 4C). An increased accumulation of LC3β-II was also found, starting at E11. LC3β-II as an indicator for lipidated LC3 and the creation of autophagosomes shows that autophagosomes are accumulated from E9 to E13 (Fig. 4A and B).

Further examination of the autophagic flux was performed by assessing the degradation of the autophagic adaptor, p62, as a complementary indicator for the measurement of degradation rate [69,70]. p62 protein degradation was diminished from E9 to E12 (Fig. 4D), with statistically significant differences at E11 and E12 (Fig. 4E). Nevertheless, the p62 value lowered at E13, reaching its detection level over the embryonic days at E9 and E10 in the cerebellar primordium (Fig. 4E). In general, based on the decreased degradation rate of autophagosomes, an accumulation of these vacuoles and inhibition of the autophagy flux from E9 to E13 have been found. Therefore, our data propose a possible association between phenotypical alterations of the cerebellar cells and autophagy flux throughout cerebellar primordium (E9−E13).

## 4 Discussion

As a result of the expression of the caudal and rostral transcription factors and extracellular signals, specifically, molecules discharged by the isthmic organizer, mouse cerebellar primordium is created in early embryonic days 7–8 [71]. As mentioned, throughout cerebellum development, coordinated generation of the multiple neural cell types in the cerebellar primordium is vital [72]. All cerebellar neurons emerge from two different germinal epithelial (neuroepithelium): the ventrally placed ventricular zone and dorsally placed rhombic lip [12,73].

This study emphasized the development of the cerebellar primordum from E9 to E13. According to the neurogenesis timeframe of the cerebellum, the PCs and CN neurons are the only post-mitotic neural populations that reside in the cerebellar primordium between E9 to E13 [10]. In the *Tgf-β*_*1*_ deficiency mouse model, an increase in neural death and microgliosis in the developing cerebellum is observed, supporting the potential role of TGF-β_1_ signaling in cerebellum primordium [15,74]. Furthermore, an experienced cellular and structural cerebella growth deficits, such as a decrease in a dendritic ramification of the PCs and smaller size cerebellum was found in TGF-β_1_ deficient mice with mutations in Tgf-β_1_ downstream effectors, including SMAD2, 3, and 4 [15].

Our data demonstrated that the expression of active TGF-β_1_ was increased at early cerebellar development, both E9 and E10, accompanied by a decrease from E11 to E13. Furthermore, the presence of principal elements of the canonical TGF-β1 signaling pathway, including TGFβR1, TGFΒR2, and cytoplasmic total and phosphorylated SMAD signaling molecules over the stated embryonic period were demonstrated. The validation of our findings by the Allen DMBA ISH analyzes showed that the total *Smad2* mRNA expression is elevated in the entire cerebellar primordium, while *Smad3* was mildly expressed in the cerebellar primordium mainly in PCP. Nevertheless, mRNA expression of both factors is diminished at E13.5. Therefore, it may be a linkage between the decrease in total SMAD2 and 3 protein levels and the stimulation of the TGF-β signaling pathway upon TGF-β1 ligand binding to the receptors’ heterotetrameric complex, resulting in the conversion of total SMAD2 and 3 to phosphorylated and activated SMADs. Further, protein expression levels of phosphorylated SMAD2 showed no signals during the primordial embryonic days at E9 and E10, with the signals appearing from E11, and reaching the greater amounts at E12 and E13. According to the quantity and accessibility of TGF-β1 ligands, the period and severity of the Smad-dependent transmitted signals are controlled [75,76]. Thus, our data underline the potential association of the Smad-dependent TGF-β signaling pathway in the creation of the PCP and positioning of the CN neurons in the cerebellar primordium from E9 to E13.

Other significant elements that control the length of time and severity of the TGF-β_1_ signaling pathway are TGFβR1 and 2 [76]. Nevertheless, the cerebellar expression of TGFβR1 and 2 over embryonic development is not known, specifically in the cerebellar primordium from E9 to E13. Based on the RT-qPCR data both *Tgfbr1* and *Tgfbr2* mRNA were expressed across the stated embryonic period. Intriguingly, the *Tgfbr1* mRNA expression level showed a decreasing trend from E9 to E13, with the highest and lowest levels at E9 and E12, respectively. Nonetheless, the *Tgfbr2* mRNA expression levels were nearly steady from E9 to E13. One possible explanation is that ligand loss in the TGF-β network happens using TGFβR2 [17]. Thus, the steady *Tgfbr2* expression level shows ongoing loss of the TGF-β_1_ ligands, contributing to the intensification and extension of the Smad-dependent signaling. Cells with defects in expression of TGFβR2, but not TGFβR1 have shown that are incapable to use up TGF-β_1_ ligands from the surroundings [17]. Our Allen DMBA ISH data revealed that *Tgfbr2* mRNA expression is not identified at chosen embryonic time points, suggesting that is a result of the loss of TGF-β_1_ ligands via TGFβR2-mediated endocytosis [17]. Thus, internalization of the TGFβR2 followed by the use and removal of the active TGF-β_1_ ligands from the cell surface could function as a main final signal that inhibits the *Tgfbr2* gene transcription and lowers the expression of the *Tgfbr2* mRNA, finally resulting in TGF-β signaling repression.

Different growth incidents are reliable on cell-cell/cell-ECM interactions, such as cell-cell adhesion and cell migration [77,78]. Tgf-βs are shown to control cell growth and differentiation via mediating the collection of ECM elements in cerebellar cortex cultured neurons, extracted from E18 Wistar rats [79]. In our study, TGF-β_1_ could upregulate the expression of a group of CAMs during early development of the cerebellar primordium. Numerous studies emphasized the significance of cadherin activity in harmonizing cellular shape, integrity, collection, and spread in different germ layers throughout morphogenesis in the mouse [32]. All these processes need close cell-cell contacts and communications interconnected by cadherins [30]. At this point, a possible control mechanism of cadherins at the transcriptional level throughout cerebellar development can be suggested, which may be regulated by the stimulated TGF-β1 signaling pathway. Furthermore, the evaluation of expression patterns of type I and type II classical cadherins, including CDH2 and CDH8, respectively was performed. Also, the expression profile of cadherin binding protein Ctnnb1 and Ncam1 was determined. Interestingly, the expression of *Cdh2* at both E11.5 and E13.5 showed upregulation and became more robust in PCP and NTZ at E13.5, based on the deduced data from the Allen DMBA ISH reference. This expression pattern explains the role of CDH2 in both post-mitotic cell counts within the cerebellar primordium over time, as they establish more intercellular associations. In our analysis, we found a steady level of *Cdh2* mRNA expression with few variations from E9 to E13, suggesting an ongoing function of the *Cdh2* gene throughout five embryonic days from E9 to E13. Contrarily to our Western blot data, where total protein expression is elevated from E9 to E11, the constant *Cdh2* mRNA expression pattern proposes the potential of translation enhancers between embryonic days 9–11, correlating with the generation time of the CN neurons and PCs in the cerebellar primordium.

The main intracellular elements connecting the extracellular ectodomains of the cadherins to the actin cytoskeleton are the catenin proteins, as reported by various studies [33,80,81]. Increased levels of cadherin/catenin combinations are present in synaptic junctions generated on both dendrites and axons of the neurons [80,81]. On this matter, the protein and mRNA expression levels of *Ctnnb1* in cerebellar primordium were determined. We have shown that even though the Ctnnb1 protein levels stayed nearly constant from E9 to E13, the *Ctnnb1* mRNA expression significantly diminished over the period, with upper and lower levels at E9 and E11/E12, respectively. Contrarily to earlier studies, we have shown that high levels of CDH2 do not impact the *Ctnnb1* mRNA expression levels from E9 to E13. This decreasing trend in *Ctnnb1* mRNA expression levels from E9 to E12, suggests that Ctnnb1 may not be a crucial intermediary of CDH2 expression in post-mitotic neural populations during their creation. Nonetheless, ISH data in Allen DMBA demonstrated a broad and steady *Ctnnb1* expression throughout the cerebellar primordium at E11.5 and E13.5, like the *Cdh2* expression pattern.

Intriguingly, the *Ctnnb1* expression pattern over time and at E13.5 becomes more compact in the PCP, and its expression reduces in the NTZ. Thus, *Ctnnb1* is required for the *Cdh2* membrane localization in the NTZ and PCP, suggesting that steady CTNNB1 protein expression would confirm the CDH2 expression and upregulation from E9 to E11. Hence, CTNNB1 activates cell-cell adhesion activity of the CDH2, resulting in the collection and unity of CN neurons and PCs in NTZ and PCP from E9 to E13. These data demonstrate the direct correlation between upregulation of both N-cadherin/CDH2 and CTNNB1 after stimulation of the TGF-β signaling pathway, associated with the period when CN neurons and PCs are imitated and assembled in the cerebellar primordium.

Further, the protein and mRNA expression levels of Cdh8 were assessed to determine the expression profile of type II cadherins in the initial developmental phases of the cerebellar primordium. A member of cadherins facilitates heterophilic cell-cell adhesions [30]. Our Western blot results validate the ISH data from Allen DMBA, demonstrating that RNA probes could not identify the *Cdh8* mRNA at E11.5 and E13.5. The differential expression patterns between type I and type II cadherins could be the potential explanation for this expression pattern [82]. In addition, the temporal expression analysis demonstrated that CDH2, a member of the type I classical cadherins, showed an increasing trend from E9 to E11, associated with lower levels from E12 to E13. On the other hand as a balance, type II cadherin-CDH8 expression starts at E12, showing a significant increase at E13. Thus, a change in the expression between CDH2 and CDH8 may control developmental processes in the cerebellar primordium from E9 to E13. Moreover, this variation displays a peak in *Cdh2* expression and an absence of *Cdh8* expression around the time of stimulation of the TGF-β signaling pathway at almost E11/E12. Hence, the stimulated TGF-β signaling pathway may upregulate the expression of type I and II cadherins, N-cadherin/CDH2, and CDH8, consecutively and transiently, correlated with proliferation, migration, and positioning of the CN neurons and PCs in NTZ and PCP, respectively.

Here, we demonstrated that the active TGF-β1 and its canonical signaling pathway are possibly associated with autophagy flux repression in cerebellar primordium during the initiation of embryonic phases. Increased creation of autophagosomes or decreased degradation was observed from E9 to E13, however, a decrease in p62 degradation also was noted at the same time. Consequently, in conclusion, the autophagosome collection was a result of a decrease in their degradation rate and possible repression of the autophagy flux at E9−E13. In addition, autophagic flux repression was not possible during the early development of the cerebellar primordium at E9 and E10, showing that autophagy is probably engaged in preparing adequate nutrients, such as amino acids, for protein synthesis and removing damaged cytoplasmic elements within the neural cells. Thereafter, great and significant levels of p62 are identified at E11 and E12, indicating the collection of autophagosomes and possible repression of the autophagy flux in the cerebellar primordium. Nonetheless, consistent with our data, the generation of the CN neurons in NTZ and PCP and placement happens with a peak at around E11.5, associated with the stimulation of Smad-dependent TGF-β signaling pathway at E11 and E12, in the cerebellar primordium. Consequently, the stimulated TGF-β signaling pathway possibly represses autophagy to facilitate neural survival by stopping the autophagy cell death and its eventual loss of mass and cells, proposing that the largest number of post-mitotic PCs and CN neurons can assemble to shape different domains within the cerebellar primordium. At E13, P62 levels diminish, and autophagic flux reverts to its normal and base levels, adequate for healthy neural cells. The impact of autophagy on the cerebellum in pathologic conditions, including long-term carbon black inhalation [83], acrylamide exposure [84], and valproic acid-induced autism [85] has been reported, which may be corresponding to inflammation and cell death in rat cerebellar cells. Also, the impact of autophagy on cerebellar development is indicated in some previous studies. Human ATG7 recessive variant, involved in degradative autophagy, induced complex neurodevelopmental diseases in the brain, muscle, and endocrine involvement causing abnormalities in the cerebellum and corpus callosum and several types of facial dysmorphism [86]. In addition, *Atg5*, *Atg7*, and *Ambra 1* are reported to contribute to embryogenesis and organ development, such as the mouse cerebellum [[87], [88], [89]]. Thus, our data identified a possible regulatory role of autophagy repression by the Smad-dependent TGF-β signaling pathway in the generation of the PCP and assembly of CN neurons in NTZ during cerebellar growth at E11 and E12.

## 5 Conclusion, limitations, and future direction

**Fig. 5:**
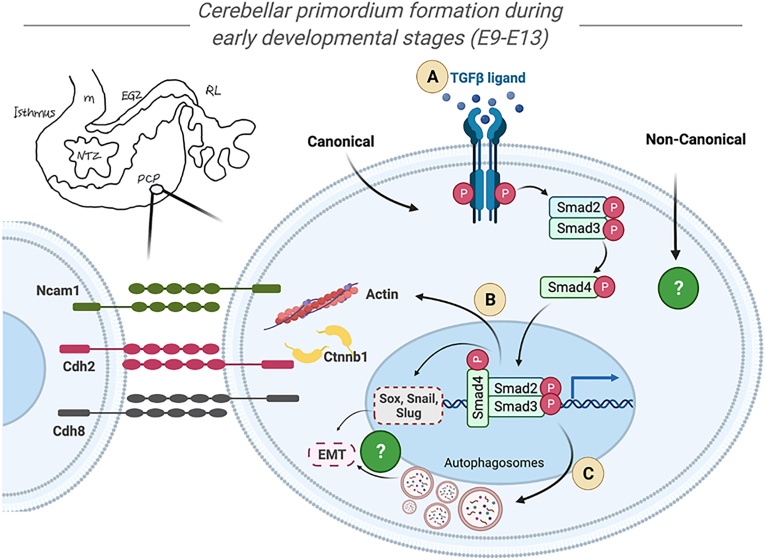
The regulatory roles of Tgfb1 in formation of the cerebellar primordium during early developmental stages at E9-E13. Based on the neurogenesis timing of the cerebellum, the only postmitotic neural populations that exist in the cerebellar primordium between E9 to E13 are the Purkinje cells and cerebellar nuclei neurons located at the Purkinje cell plate and nuclear transitory zone, respectively. Expression of active Tgfb1 is intensely high at earliest embryonic days at both E9 and E10, followed by a substantial reduction in its value from E11 to E13. Our study shows that Tgfb1 through the canonical TGF-β signaling pathway could upregulate the expression of CAMs. On the other hand, activation of the Tgf-β signaling pathway and its consequent reduction in available Tgfb1 amounts, autophagic-flux, which the Tgf-β1 regularly induces, is inhibited from E9 to E13. **A**) Activation of the Smad-dependent Tgf-β1 signaling pathway in the mouse cerebellar primordium at E9-E13; Our finding showed the presence of main components of the canonical Tgf-β signaling pathway, including Tgfbr1, Tgfbr2and cytoplasmic total and phosphorylated Smad signaling molecules during the given embryonic period. **B**) Tgfb1 upregulates the expression of Cadherins in the mouse cerebellar primordium at E9-E13; Tgfb1 by upregulating the expression of cell adhesion proteins, including cdh2, Cdh8, Ncam, and cadherin binding protein β-catenin (Ctnnb1), contribute to the physical interactions and connectivity between postmitotic Purkinje cells and cerebellar nuclei neurons, locating in the Purkinje cell plate and nuclear transitory zone during earliest developmental stages from E9 to E13 with a peak at E11. These data show the direct association between upregulation of both Cdh2 and Ctnnb1, Cdh8 and Ncam upon activation of the Tgf-β signaling pathway, correlated to time points during which Purkinje cells and cerebellar nuclei neurons are born, migrating and positioning in cerebellar primordium. **C**) Tgfb1 inhibits autophagic-flux in the mouse cerebellar primordium at E9-E13; Tgfb1, by regulating the autophagic-flux, responds to the extracellular signals such as stress (starvation, hypoxia, aggregation of unwanted materials) to maintain cellular homeostasis and provide neural protection during cerebellar development. The results from our study pinpoint a potential regulatory role of autophagy inhibition by Smad-dependent Tgf-β signaling pathway in the formation of the Purkinje cell plate and accumulation of cerebellar nuclei neurons in nuclear transitory zone during early cerebellar development.

This is the first extensive developmental-based effort to assess the expression of the elements of the TGF-β signaling pathway and its regulatory impacts on cadherins expression and autophagy in cerebellar primordium during the initiation of embryonic days from E9 to E13. TGF-β signaling pathway showed important roles during proliferation, migration, and positioning of the CN neurons and PCs in NTZ and PCP, respectively (summarized in Fig. 5).

Even though we tried to optimize the study, some potential limitations exist. As a consequence of the small size of the embryo and specifically the cerebellar primordium at E9/E10, a potential small percentage of error may happen, upon removal of the cerebellar primordium. Moreover, the inclusion of the smallest size in the study was limited to the E9 embryo. Thus, we were unable to screen the episodes of the establishment of cerebellar primordium (E7−E8.5) and explain the underlying mechanisms that happen before E9. Furthermore, Allen DMBA ISH data only demonstrates the allocation of the mRNA expression of the *Tgf-β* signaling pathway, at E11.5 and E13.5, so it does not include the cerebellar primordium at E9/E10. In addition, ISH data are not specific between different cell types, and further validation is needed to visualize the protein expression of all the markers by immunohistochemistry and to identify their colocalization in the target cells by immunofluorescent.

For the future direction of this study, more tests are required to answer some important questions. First, it is worth assessing the stimulation and involvement of non-canonical TGF-β signaling pathways, including MAP kinase, PI3K/Akt, and Rho-like GTPase, during cerebellar development. Second, the underlying mechanism in which TGF-β upregulates the expression of the cadherins should be further investigated. Otherwise stated, clarification is needed on whether TGF-β directly and through the SMAD family of transcription factors upregulate the expression of *cadherin* genes or *Tgf-β* by imitating the process, in which EMT is activated to reprogram cadherin gene expression. Finally, it is essential to approve the autophagy flux rate and involvement of TGF-β pathways and their functions in positioning the neuronal types and distribution by specific neuronal markers and using proper autophagy flux inihbtors (Bafilomycin or chloroquine). This may pave the way to a better understanding of cerebellar development in health and diseases.

## Conflicts of interest

The authors have no conflicts of interest.

## Data Availability

All raw data for WB have been included as supplementary information.
