# Supplementary material for: Potential role of TGFΒ and autophagy in early crebellum development
Source: Biochem Biophys Rep. 2022 Oct 3;32:101358. doi: 10.1016/j.bbrep.2022.101358 (PMC9535406; doi:10.1016/j.bbrep.2022.101358)
Supplement: Multimedia component 1 [file mmc1.pptx]

## Slide 1
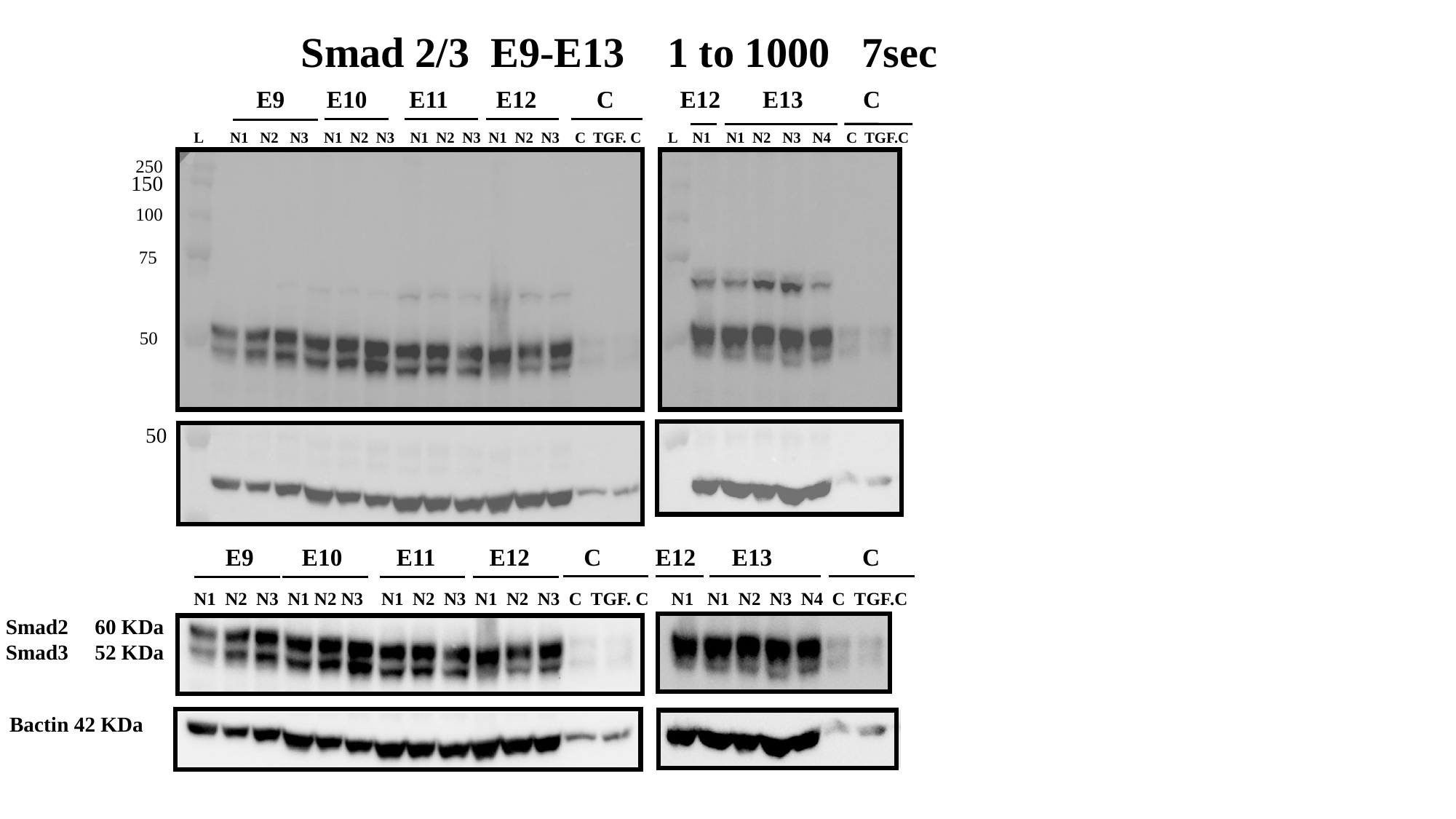

# Smad 2/3 E9-E13 1 to 1000 7sec
 E9 E10 E11 E12 C E12 E13 C
L N1 N2 N3 N1 N2 N3 N1 N2 N3 N1 N2 N3 C TGF. C L N1 N1 N2 N3 N4 C TGF.C
250
150
100
75
50
50
 E9 E10 E11 E12 C E12 E13 C
N1 N2 N3 N1 N2 N3 N1 N2 N3 N1 N2 N3 C TGF. C N1 N1 N2 N3 N4 C TGF.C
 Smad2 60 KDa
 Smad3 52 KDa
Bactin 42 KDa

## Slide 2
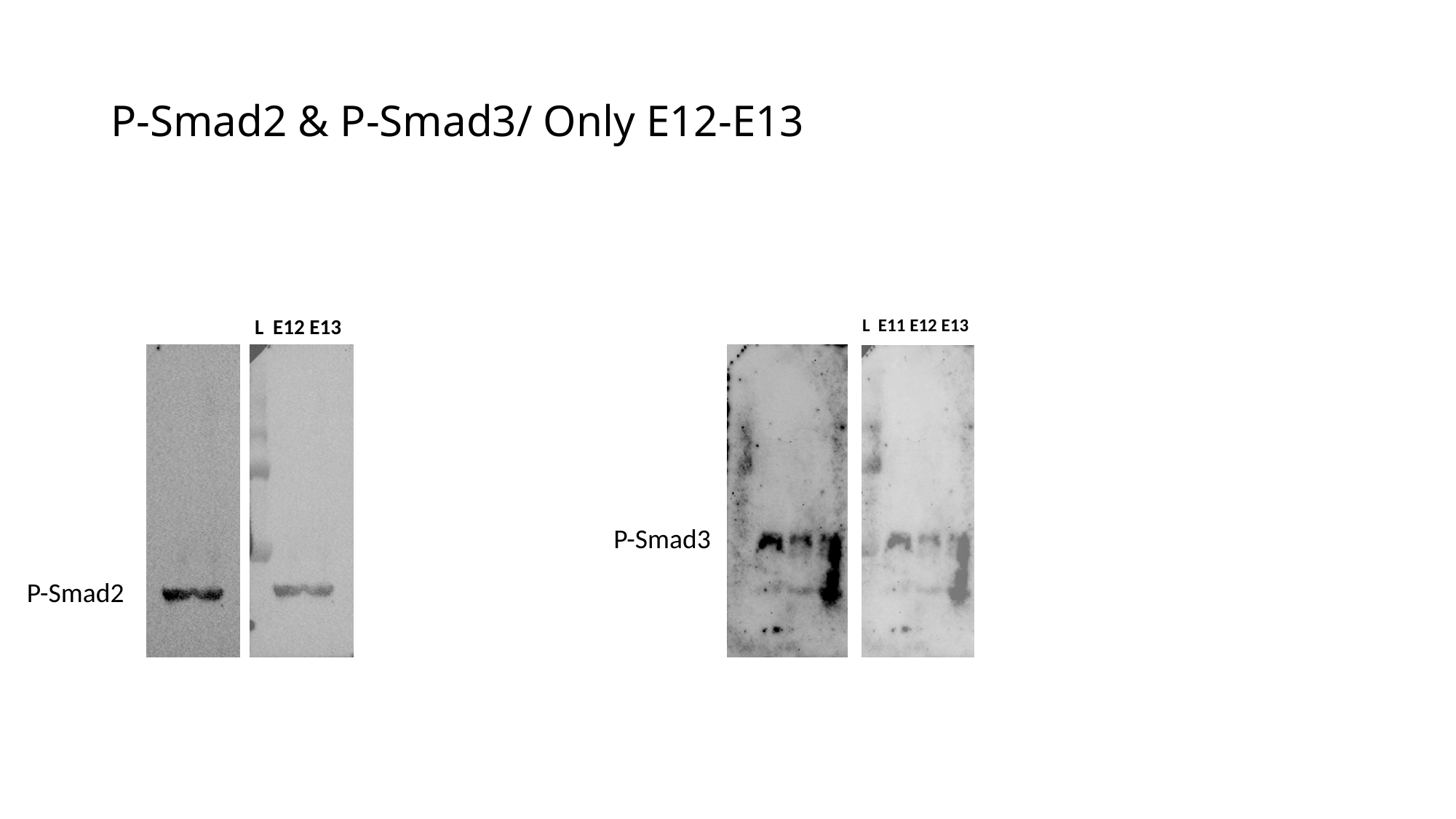

# P-Smad2 & P-Smad3/ Only E12-E13
L E12 E13
L E11 E12 E13
P-Smad3
P-Smad2

## Slide 3
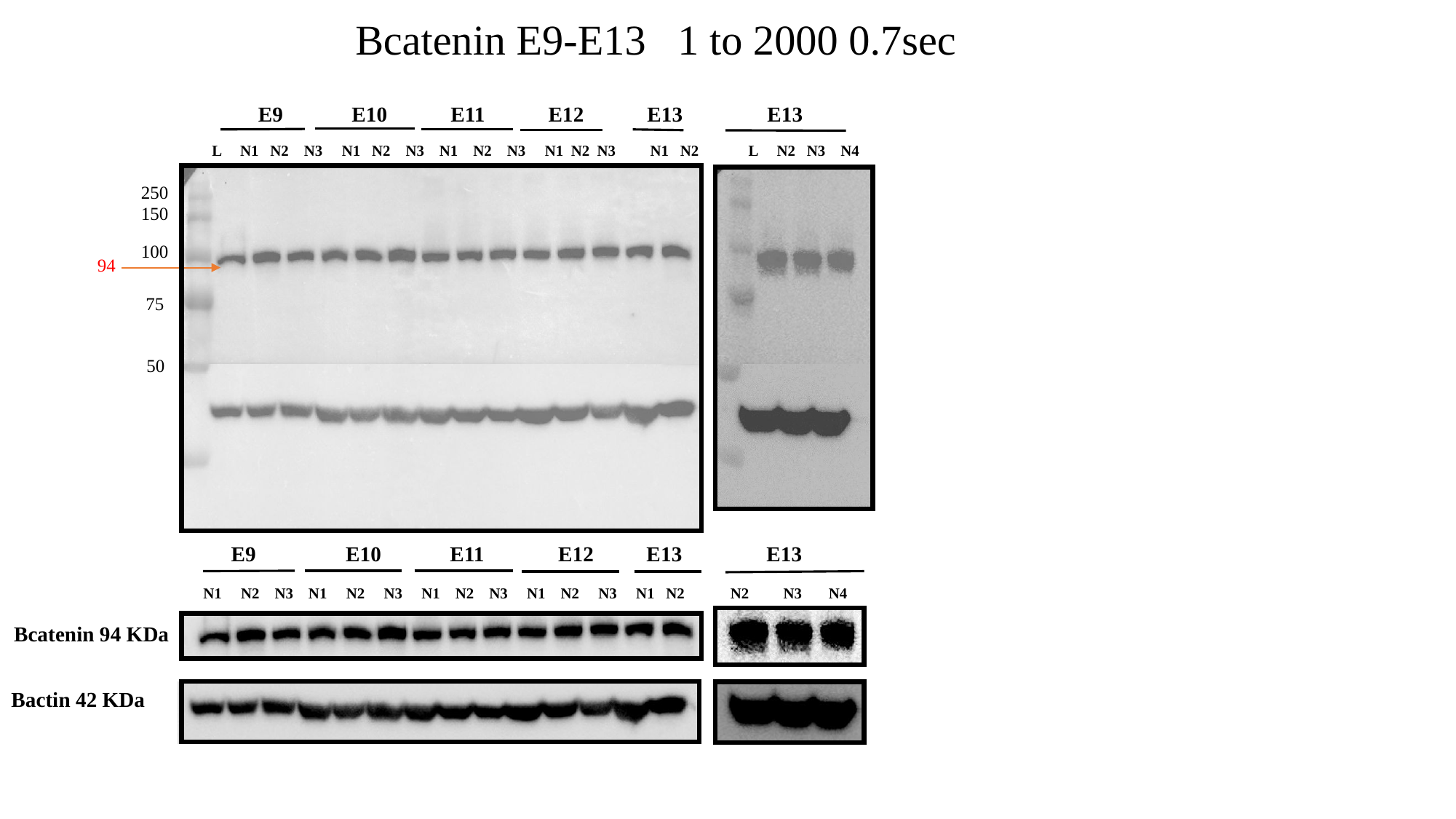

# Bcatenin E9-E13 1 to 2000 0.7sec
E9 E10 E11 E12 E13 E13
 L N1 N2 N3 N1 N2 N3 N1 N2 N3 N1 N2 N3 N1 N2 L N2 N3 N4
250
150
100
94
75
50
E9 E10 E11 E12 E13 E13
 N1 N2 N3 N1 N2 N3 N1 N2 N3 N1 N2 N3 N1 N2 N2 N3 N4
Bcatenin 94 KDa
Bactin 42 KDa

## Slide 4
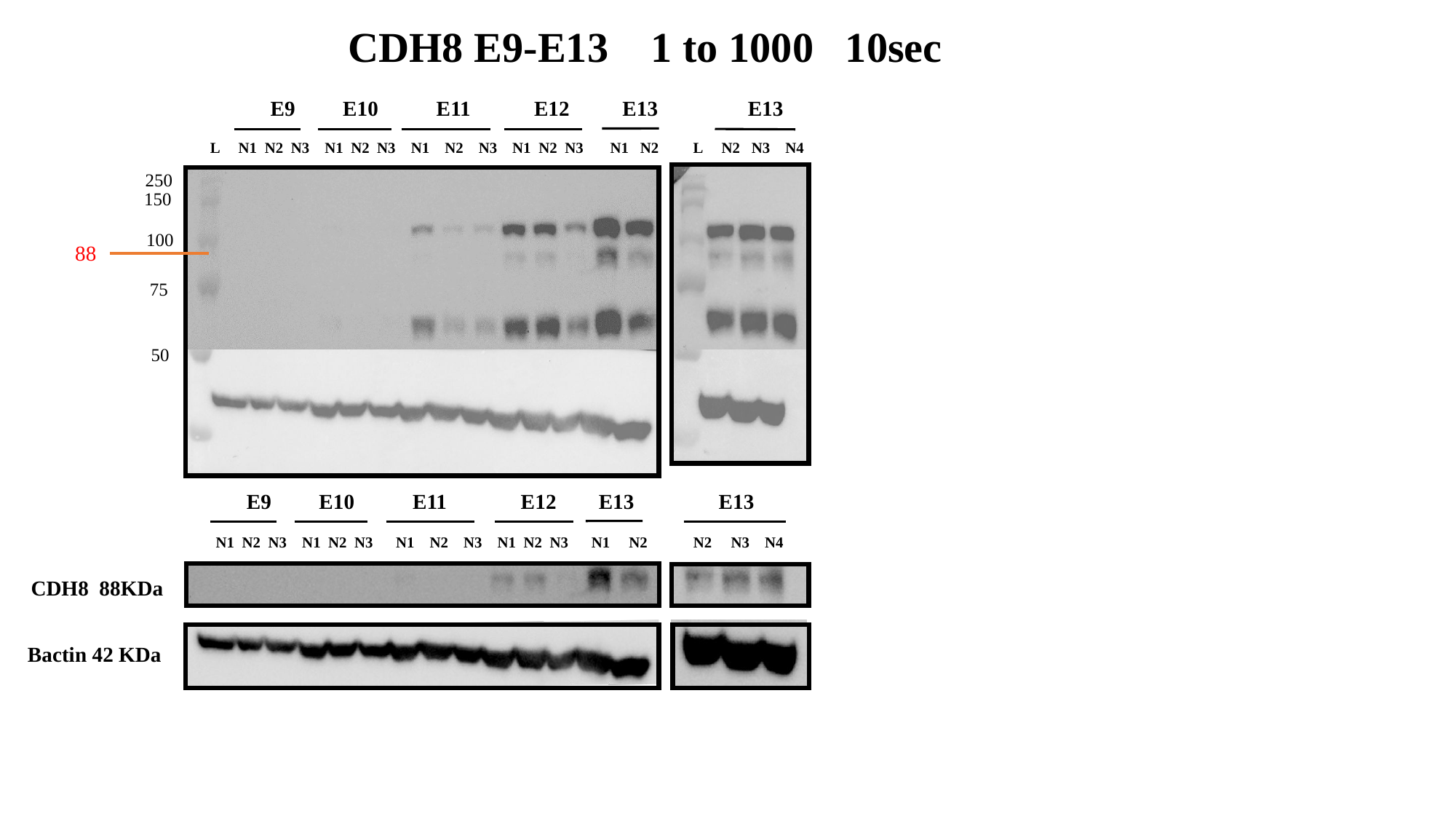

# CDH8 E9-E13 1 to 1000 10sec
E9 E10 E11 E12 E13 E13
L N1 N2 N3 N1 N2 N3 N1 N2 N3 N1 N2 N3 N1 N2 L N2 N3 N4
250
150
100
88
75
50
E9 E10 E11 E12 E13 E13
 N1 N2 N3 N1 N2 N3 N1 N2 N3 N1 N2 N3 N1 N2 N2 N3 N4
CDH8 88KDa
Bactin 42 KDa

## Slide 5
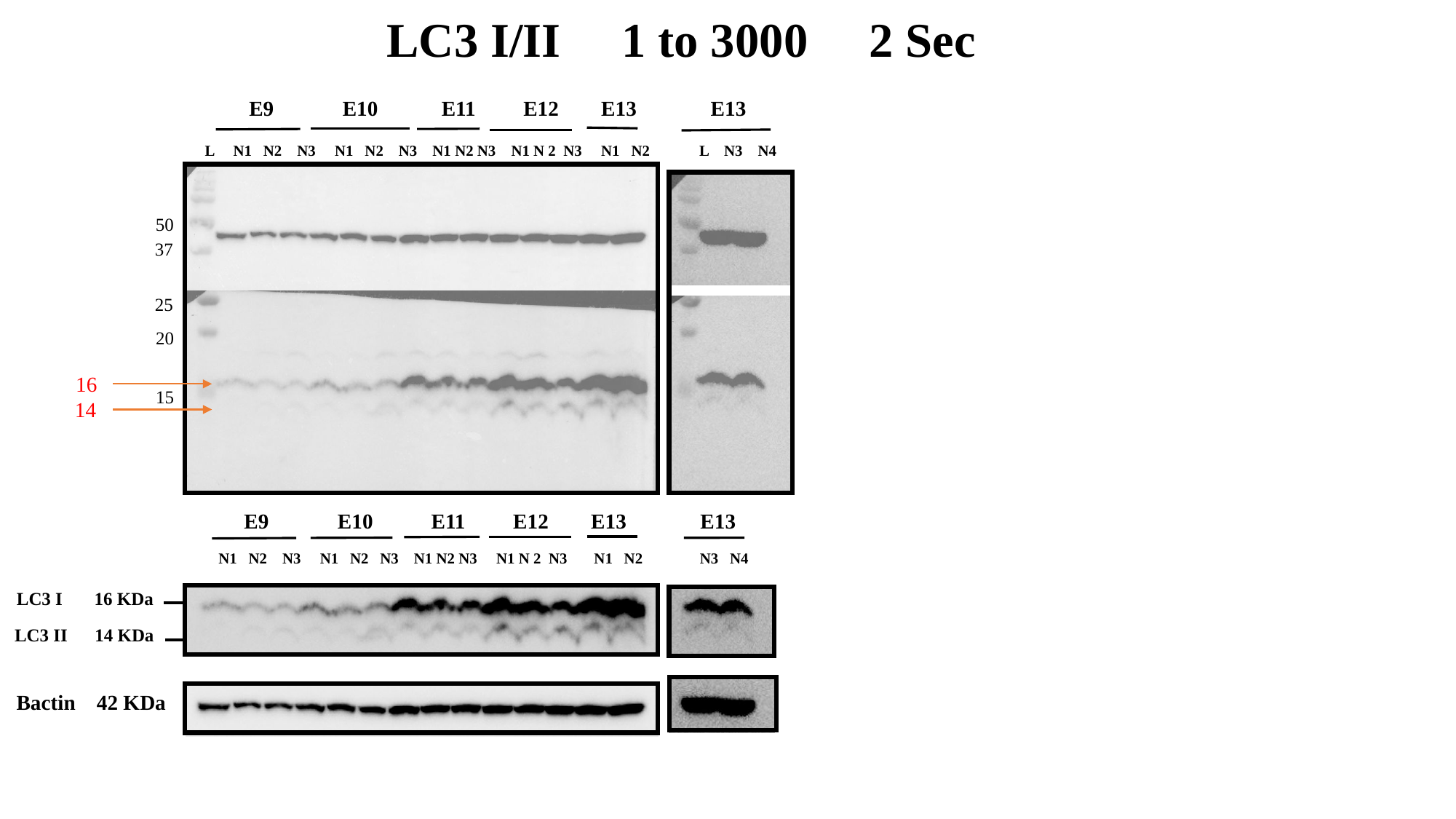

# LC3 I/II 1 to 3000 2 Sec
E9 E10 E11 E12 E13 E13
 L N1 N2 N3 N1 N2 N3 N1 N2 N3 N1 N 2 N3 N1 N2 L N3 N4
50
37
25
20
16
15
14
E9 E10 E11 E12 E13 E13
 N1 N2 N3 N1 N2 N3 N1 N2 N3 N1 N 2 N3 N1 N2 N3 N4
LC3 I 16 KDa
LC3 II 14 KDa
Bactin 42 KDa

## Slide 6
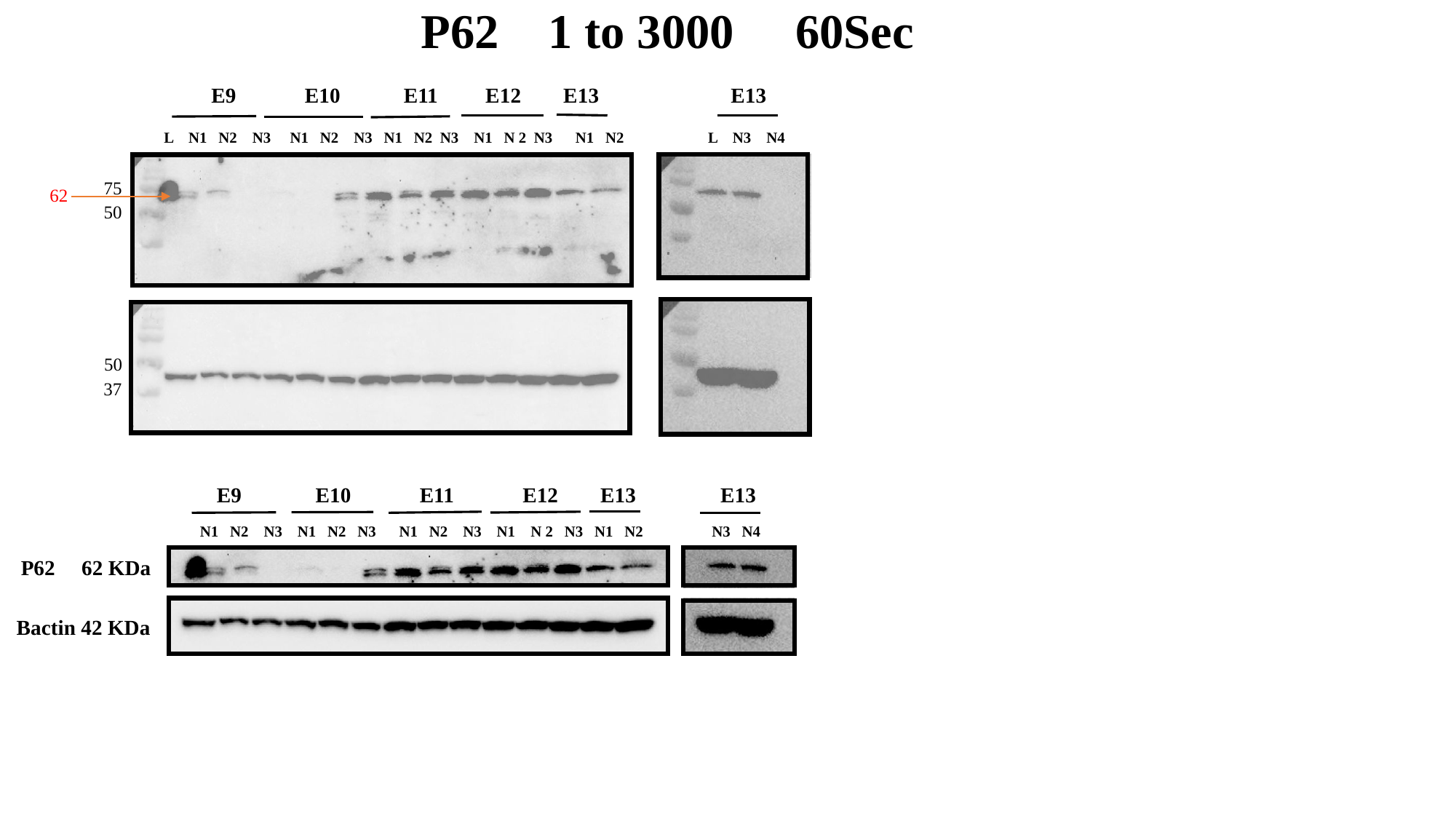

# P62 1 to 3000 60Sec
E9 E10 E11 E12 E13 E13
 L N1 N2 N3 N1 N2 N3 N1 N2 N3 N1 N 2 N3 N1 N2 L N3 N4
75
62
50
50
37
E9 E10 E11 E12 E13 E13
 N1 N2 N3 N1 N2 N3 N1 N2 N3 N1 N 2 N3 N1 N2 N3 N4
P62 62 KDa
Bactin 42 KDa

## Slide 7
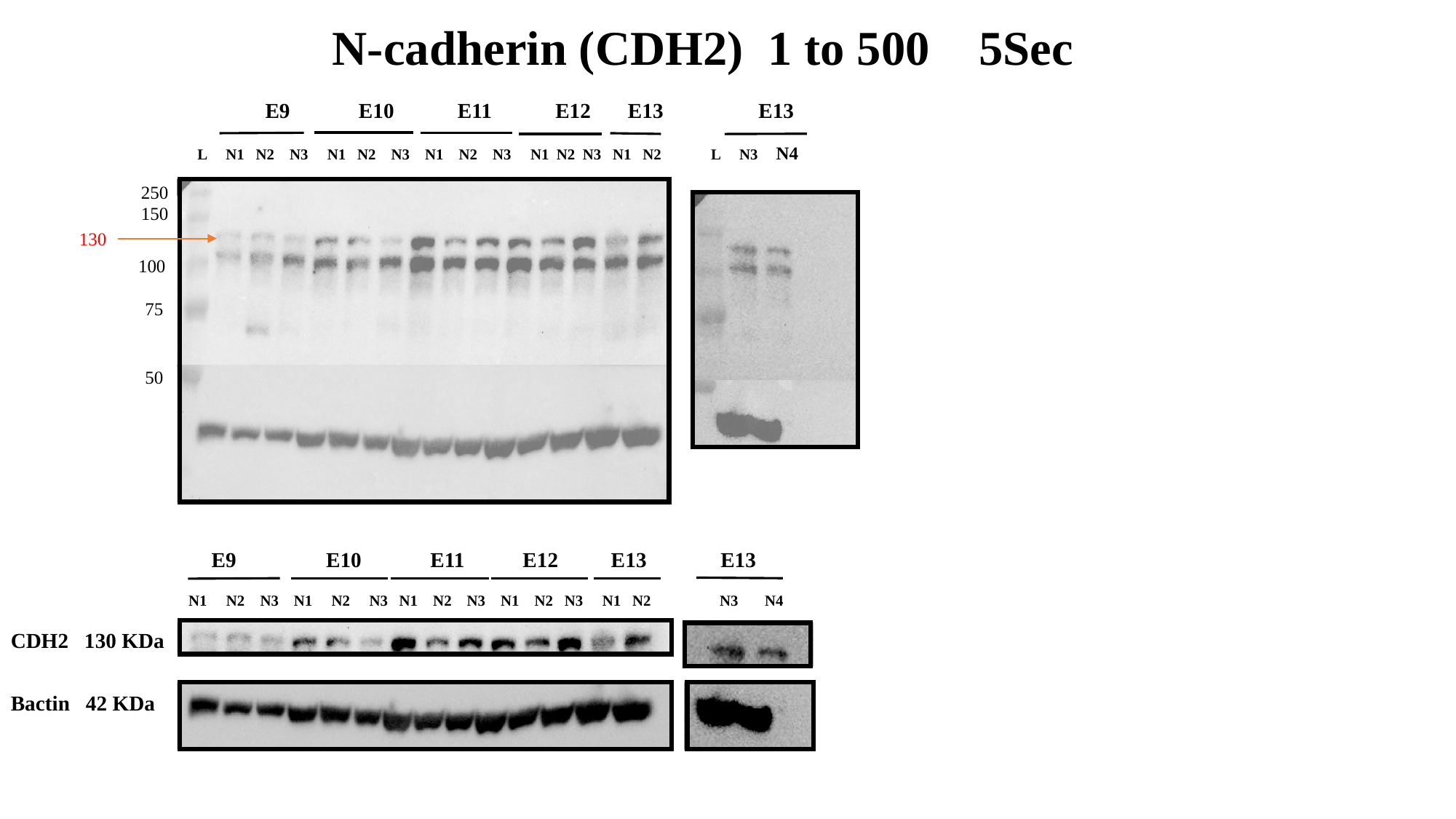

# N-cadherin (CDH2) 1 to 500 5Sec
E9 E10 E11 E12 E13 E13
 L N1 N2 N3 N1 N2 N3 N1 N2 N3 N1 N2 N3 N1 N2 L N3 N4
250
150
130
100
75
50
E9 E10 E11 E12 E13 E13
N1 N2 N3 N1 N2 N3 N1 N2 N3 N1 N2 N3 N1 N2 N3 N4
CDH2 130 KDa
Bactin 42 KDa

## Slide 8
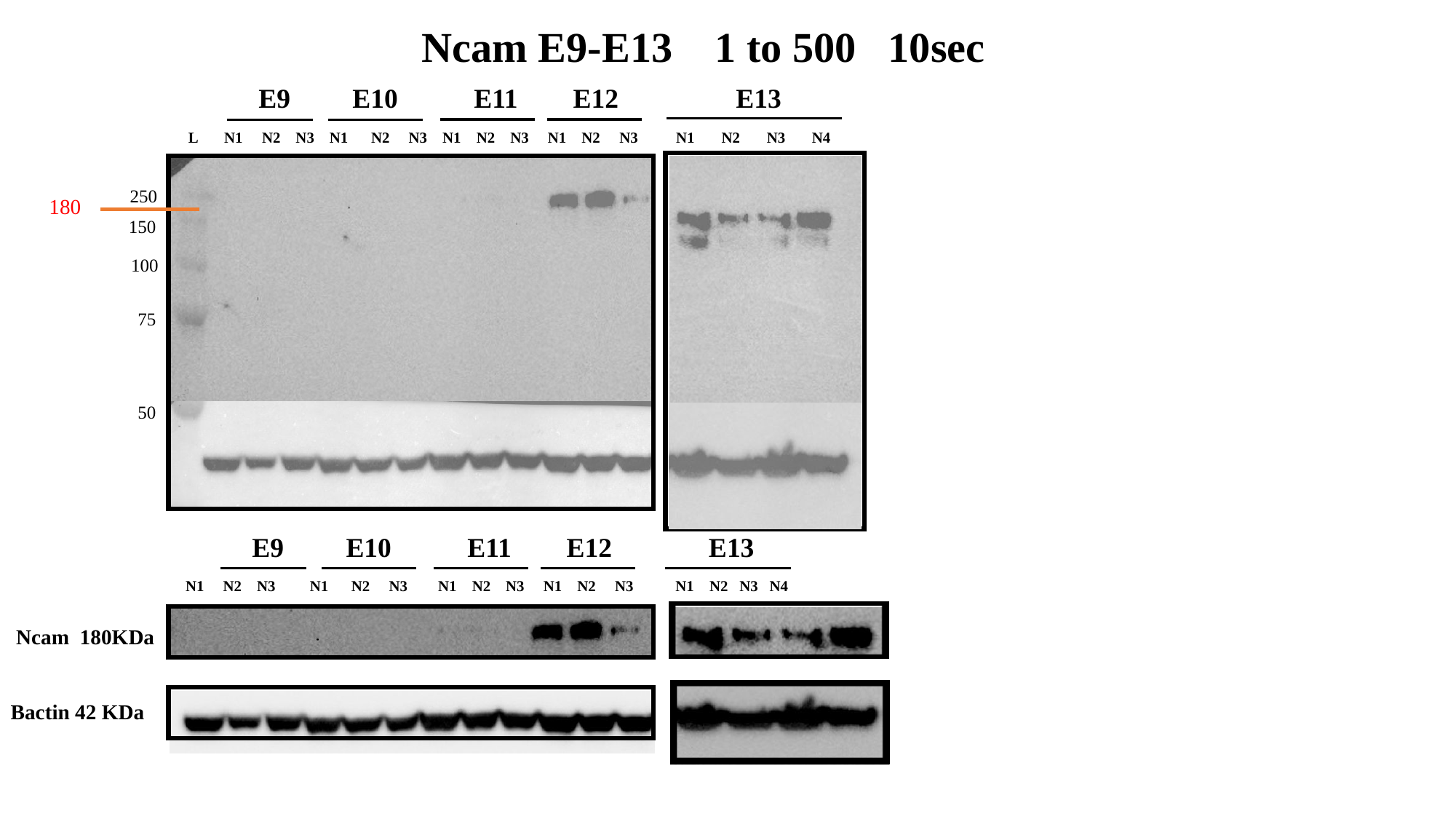

# Ncam E9-E13 1 to 500 10sec
 E9 E10 E11 E12 E13
L N1 N2 N3 N1 N2 N3 N1 N2 N3 N1 N2 N3 N1 N2 N3 N4
250
180
150
100
75
50
 E9 E10 E11 E12 E13
 N1 N2 N3 N1 N2 N3 N1 N2 N3 N1 N2 N3 N1 N2 N3 N4
 Ncam 180KDa
Bactin 42 KDa
